# ACADL-YAP axis activity in non-small cell lung cancer carcinogenicity

**DOI:** 10.1186/s12935-024-03276-7

**Published:** 2024-02-24

**Authors:** Kegong Chen, Chunqiao Hong, Weibo Kong, Guanghua Li, Zhuang Liu, Kechao Zhu, Chen Lu, Panpan Si, Pan Gao, Guangyao Ning, Renquan Zhang

**Affiliations:** 1 Department of Thoracic Surgery, The First Affiliated Hospital of Anhui Medical University, No. 218, Jixi Road, Shushan District, Hefei, 230001 China; 2 Key Laboratory of Respiratory Disease Research and Medical Transformation of Anhui Province, Hefei, 230001 China; 3 Department of Critical Care Medicine, Xiamen Chang Gung Hospital Hua Qiao University, Xiamen, 361013 China; 4 Departments of Thoracic Surgery, Anhui Provincial Chest Hospital, Hefei, 230001 China; 5 Department of Thoracic Surgery, The Second Affiliated Hospital of Harbin Medical University, Harbin, 150000 China; 6 Department of Cardiovascular Surgery, The First Affiliated Hospital of Anhui Medical University, Hefei, 230001 China; 7 Department of Urology, The First Affiliated Hospital of Anhui Medical University, Hefei, 230001 China

**Keywords:** Non-small cell lung cancer, Nuclear transfer, Phosphorylation, YAP, ACADL

## Abstract

**Background:**

The role of Acyl-CoA dehydrogenase long chain (ACADL) in different tumor types had different inhibiting or promoting effect. However, its role in non-small cell lung cancer (NSCLC) carcinogenicity is not clear.

**Method:**

In this study, we utilized The Cancer Genome Atlas (TCGA) database to analyze ACADL expression in NSCLC and its correlation with overall survival. Furthermore, we investigated the function of ACADL on cellular proliferation, invasion, colony, apoptosis, cell cycle in vitro with NSCLC cells. Mechanistically, we evaluated the regulatory effect of ACADL expression on its downstream factor yes-associated protein (YAP) by assessing YAP phosphorylation levels and its cellular localization. Finally, we verified the tumorigenic effect of ACADL on NSCLC cells through xenograft experiments in vivo.

**Results:**

Compared to adjacent non-cancerous samples, ACADL significantly down-regulated in NSCLC. Overexpression of ACADL, effectively reduced the proliferative, colony, and invasive capabilities of NSCLC cells, while promoting apoptosis and inducing cell cycle arrest. Moreover, ACADL overexpression significantly enhanced YAP phosphorylation and hindered its nuclear translocation. However, the inhibitory effect of the overexpression of ACADL in NSCLC cells mentioned above can be partially counteracted by YAP activator XMU-MP-1 application both in vitro and in vivo.

**Conclusion:**

The findings suggest that ACADL overexpression could suppress NSCLC development by modulating YAP phosphorylation and limiting its nuclear shift. This role of ACADL-YAP axis provided novel insights into NSCLC carcinogenicity and potential therapeutic strategies.

**Supplementary Information:**

The online version contains supplementary material available at 10.1186/s12935-024-03276-7.

## Introduction

85% of lung cancer is composed of non-small cell lung cancer (NSCLC) which is characterized by high incidence and mortality rates [1, 2]. About 3/4 of NSCLC patients are diagnosed with a condition that cannot be completely removed by surgery [3, 4]. Despite the advance of chemotherapy, targeted therapy (like epidermal growth factor receptor (EGFR), anaplastic lymphoma kinase), and immunotherapy (programmed death-ligand 1 inhibitors) strategies [5, 6], the prognosis of NSCLC is still not satisfactory [7]. According to the statistics, the overall survivals of 5-year of NSCLC stage I, stage II, stage III, stage IV are 47, 30, 10%, and less than 1%, respectively [8, 9]. Although anti-programmed cell death 1 (PD1)/programmed cell death ligand 1 (PD-L1) immunotherapy has emerged as a standard of care for stage III-IV NSCLC patients, it cannot be ignored that there are still part patients who do not respond to immunotherapy, which may be due to complex tumor heterogeneity or some molecular mechanisms that have not yet been elucidated [10, 11]. Therefore, continuing to deeply reveal the pathogenic molecular mechanisms of NSCLC is beneficial for providing theoretical reference for personalized treatment in the future.

The catalysis of β-oxidation of long-chain fatty acyl-CoAs is primarily driven by Acyl-CoA dehydrogenase long chain (ACADL) during fatty acid oxidation in the mitochondria [12, 13]. ACADL is widely involved in energy and phospholipid metabolism, and its abnormal expression has been detected in various conditions, including cardiovascular disease, central obesity, nonketotic hyperglycemia, and diabetes [14–17]. Moreover, the role of ACADL in human tumors has also been gradually recognized. For instance, in esophageal squamous cell carcinoma (ESCC), ACADL acts as an oncogene and its expression is negatively correlated with prognosis [18]. Similarly, ACADL contributes to the progression and malignancy of prostatic carcinoma [19]. However, in hepatocellular carcinoma (HCC), ACADL expression is reduced and has a suppressive effect on cancer, with lower levels of ACADL associated with poor outcomes [20]. Meanwhile, immune checkpoint PD1 and PDL1, as accomplices, can help tumor cells detach from immune cell surveillance in NSCLC, thereby promoting tumor progression. A previous study suggested ACADL could inhibit the PDL1 transcription and promote the tumor cell survival in chemotherapeutic process [21]. These studies suggest that the expression status of ACADL may exert different effects on tumor progression. However, there is limited research on the role and efficacy of ACADL in NSCLC.

Yes-associated protein (YAP) is initially reported to be responsible for mammalian lung development and lung tissue regeneration and repair by controlling the balance between its cytoplasmic and nuclear localization [22]. However, recent studies suggest that excessive nuclear YAP could interact with the transcriptional activator with PDZ-binding motif (TAZ), serving as transcriptional co-activators of oncogenic genes to participate in the expression of oncogenic genes [23–25]. Moreover, in NSCLC, YAP activity is contributed to cell proliferation and metastasis while inhibiting YAP activity limits above phenomenon [26, 27]. In addition, increased YAP expression and nuclear localization are responsible for EGFR inhibitors resistance [28]. In related meta-analysis, high expression level of YAP is associated with progression of NSCLC and is identified as an independent factors for poor prognosis [29]. And YAP is also a regulator of PDL1 in NSCLC (29,383,103). The above results suggest YAP is an exact factor in NSCLC development but it is currently unclear whether YAP can be regulated by ACADL.

In our study, we aimed to explore the expression of ACADL in NSCLC patients and understand whether ACADL can participate in the carcinogenicity of NSCLC by regulating the expression and nuclear localization of YAP in vitro and in vivo studies. The results would highlight the mechanism of action of ACADL in regulating the development of NSCLC, and provide certain theoretical reference for future gene therapy targeting ACADL.

## Materials and methods

### Detection of ACADL expression in NSCLC

A total of 515 profiles of lung adenocarcinoma (LUAD) and 59 paracancer samples, and 501 profiles of lung squamous cell carcinoma (LUSC) and 49 paracancer specimens were obtained from the Cancer Genome Atlas (TCGA) database. (https://portal.gdc.cancer.gov/) followed by being annotated with HUGO annotation file [30]. Differential expression analyses between LUAD and its paracancerous tissues, LUSC, and paracancerous tissues, as well as NSCLC and its paracancerous tissues, were performed using limma package in R software. And the differential expression of ACADL between the above groups was displayed using violin plot. Additionally, the role of ACADL in NSCLC survival was evaluated using the online tool called: Kaplan-Meier plotter (http://kmplot.com/analysis/), which incorporates mRNA‐seq and microarray profiles from different tumors, along with overall survival information collected from TCGA, Gene Expression Omnibus as well as Cancer Biomedical informatics Grid databases [31]. The protein expression of ACADL in LUAD, LUSC and normal lung tissues were investigated with The Human Pathology Atlas database (https://www.proteinatlas.org) which collects various proteins expression in major cancer and normal tissue types verified by immunohistochemistry (IHC) [32]. There is a total of 3 normal lung tissues, 10 LUSC samples, and 12 LUSC samples. And all the sections were evaluated and recorded as “Not detected”, “Low”, “Medium” and “High”. Here, the sections were quantified as: 1 = Not detected; 2 = Low; 2 = Medium and 4 = High.

### Culture and treatment of NSCLC cells

The RPMI-1640 medium (Gibco, USA) containing with 10% fetal bovine serum (Gibco, USA) was utilized for culture of NSCLC cell lines H292, H1944, H1299 and A549 at a 37 °C incubator with 5% CO_2_ atmosphere. A lentiviral vector carrying the full-length open reading frame of ACADL was obtained from GenePharma Corporation (China) and was utilized to construct cell lines that stabilized overexpressing ACADL in H1299 and A549. Lentiviral transfection was performed according to the user guidelines with a multiplicity of infection as 20. In addition, for ACADL knock out (KO-ACADL) in H292 and H1944, a ACADL CRISPR/Cas9 KO plasmid, and its corresponding scrambled control plasmid (KO-SCR), designed and constructed by Genomeditech (Shanghai, China), were utilized for cell transfection with lipofectamine 2000 according to the manufacturer’s instructions. And a pcDNA 3.1 plasmid containing the full-length open reading frame of ACADL was obtained from GenePharma Corporation (China) and was utilized to rescues ACADL expression in KO-ACADL cells with lipofectamine 2000. Follow-up experiments were conducted 48 h after transfection. The YAP activator, XMU-MP-1 (1 mM, Selleckchem, USA), and an equal volume of DMSO were used to treat the transfected cell for 24 h before the follow-up assays.

### Western blot

The western blot assay was performed following the previously reported method [33]. The protein samples were extracted with RIPA solution (Beyotime Institute of Biotechnology, China) supplemented with PMSF (1 mM, Beyotime Institute of Biotechnology, China). Nuclear proteins were isolated using the nuclear and cytoplasmic protein extraction kit according to the manufacturer’s instructions (Beyotime Institute of Biotechnology, China). Primary antibodies used were as follows: ACADL (dilution: 1:1000, cat.no: HPA011990, Sigma-Aldrich, USA), P21 (dilution: 1:1,000, cat.no: 2947, CST, USA), P27 (dilution: 1:1,000, cat.no: 3698, CST, USA), Cyclin B1 (dilution: 1:1,000, cat.no: 4135, CST, USA), Cyclin D1 (dilution: 1:1,000, cat.no: 55,506, CST, USA). YAP (dilution: 1:1,000, cat.no: 14,074, CST, USA), YAP (Ser127) (dilution: 1:1,000, cat.no: 13,008, CST, USA), GAPDH (dilution: 1:5,000, cat.no: 60004-1-Ig, Proteintech, China), Histone H3 (dilution: 1:1000; cat.no: ab1791, Abcam, USA) β-actin (dilution: 1:1000, cat.no: 60,008, Proteintech, China). The HRP labeled anti-IgG antibody (dilution: 1:20,000, cat.no: ZB-2306; ZSGB-BIO, China) was used to combine the primary antibodies. The bands were visualized using the ECL reagent (WBKLS0500, Millipore) and captured using the Gel Dock™ XR + system with the ChemiDoc MP System (Bio-Rad). The quantitative analysis of the western blot assay was performed using ImageJ.

### Quantitative reverse transcription polymerase chain reaction (qRT-PCR)

According to the corresponding manufacturer’s instructions, the RNA samples of cells were harvested with Hipure Total RNA Mini Kit II (Magen, China), followed by transcription with PrimeScript RT Reagent Kit (TaKaRa, Japan). Subsequently, qPCR was implemented via the kit from SYBR Premix Ex Taq II (TaKaRa, Japan). The cycling conditions were set as follows: Preheating at 95˚C for 10 min, followed by 45 cycles at 95˚C for 15 s, 60˚C for 30 s, and 72˚C for 30 s. The primer sequences for the target genes were provided in Table 1. The expression of target genes was normalized to GADPH with 2^−ΔΔct^ formula.

**Table 1:** Primers sequences used in the RT-qPCR experiments

Gene	Forward primer (5’- 3’)	Reverse primer (5’- 3’)
CTGF	GGGAAATGCTGCGAGGAGT	CTTCCAGTCGGTAAGCCGC
CYR61	GAAGCGGCTCCCTGTTTTTG	CGGGTTTCTTTCACAAGGCG
ANKRD1	AGAACTGTGCTGGGAAGACG	GCCATGCCTTCAAAATGCCA
GAPDH	GCACCGTCAAGGCTGAGAAC	TGGTGAAGACGCCAGTGGA

RT-qPCR: Reverse transcription-quantitative polymerase chain reaction. CTGF: Connective tissue growth factor, CYR61: Cysteine rich angiogenic inducer 61, ANKRD1: Ankyrin repeat domain 1

### IHC and immunofluorescence (IF) staining

For IHC stating, antigen retrieval was performed by incubating the slices in citrate buffer at 100 °C for 3 min. Nonspecific epitopes were blocked with 3% BSA at room temperature for 30 min. Subsequently, the primary antibodies, including Ki67 (dilution: 1:200; ab15580, Abcam, USA), epithelial cadherin (E-cadherin) (dilution: 1:500; cat.no: ab40772, Abcam, USA), and proliferating cell nuclear antigen (PCNA) (dilution: 1:100; cat.no: ab92552, Abcam, USA), were utilized for incubating the tissue slices at 4˚C overnight. The HRP labeled anti-IgG antibody (cat.no: ZB-2306; ZSGB-BIO, China) was then applied and incubated at room temperature for 1 h. Visualization and nuclei staining were achieved using DAB (ZSGB-BIO, China) and hematoxylin (Solarbio, China) for 3 and 15 min, respectively. Lastly, images were acquired using a light microscope (OLYMPUS, Japan). And the immunohistochemical intensity was analyzed based on the mean integrated optical density (mean IOD) for expression of target proteins by Image J.

For IF staining, the cells inoculated in a 24-well plate were fixed with 95% ethanol for 20 min, permeabilizated by 0.2% Triton X-100 for 30 min and blocked with 3% BSA for 30 min. Next, the cells were incubated with the YAP antibody (dilution: 1:200, cat.no: 14,074, CST, USA) overnight at 4 °C. After removing the primary antibody, the cells were treated with IgG H&L antibody (cat.no: ab150079, Abcam, USA) for 1 h at room temperature. Finally, the nucleus was stained with DAPI (Solarbio, China) and observed under a fluorescence microscope (OLYMPUS, Japan).

### Apoptosis detection

The apoptosis level of cells was evaluated with Annexin V-FITC Apoptosis Detection Kit (Sigma, USA) according to the official protocols. the cells were subjected to flow cytometry (BD Pharmingen, USA) and analyzed by FlowJo software.

### Colony formation assay

The cells with a density of 4 × 10^3^ cells/well were cultured in the 6-well plate and continuously cultured for 14 days followed by being fixed with 4% paraformaldehyde (Biosharp, China). Subsequently, the colonies were visualized by incubating them with 0.1% crystal violet at room temperature for 30 min. Colonies consisting of more than 50 cells were imaged using a camera and analyzed by using ImageJ.

### Cell proliferation assay

For assessing cellular proliferation, cells were plated in 96-well plates at a density of 5 × 10^3^ cells/well and cultured for an additional 72 h. The cellular activity was evaluated using the 5-ethynyl-2’-deoxyuridine (EdU) assay kit (Solarbio, China) following the official protocol. The images were captured with fluorescent microscope (Leica, Germany) and analyzed using ImageJ software.

### Transwell assay

The transwell assay was utilized to evaluate the migration ability of cells. Briefly, cells at a density of 1 × 10^5^/well were seeded in the upper chambers (Coring, USA), which pre-coated with Matrigel (BD, USA), with serum‑free RPMI-1640 medium. The bottom chambers were loaded with 600 µl medium containing 10% FBS. After culture for 24 h, cells on the upper surface were scraped off with a cotton ball. And cells on the inner surface were fixed with 4% paraformaldehyde and stain with 0.1% crystal violet. The images were captured with a light microscope (OLYMPUS, Japan). Quantitative analysis of migrated cells was performed using ImageJ.

### Tumor growth xenograft model

All animal procedures were performed in accordance with the Guide for the Care and Use of Laboratory Animals published by the National Institutes of Health of the United States. The experimental protocols were approved by the Experimental Animal Ethics Committee of Anhui Medical University (grant.no: LLSC20211508). Male nude mice aged 6–8 weeks old (18∼22 g) were obtained from the Experimental Animal Center of Anhui Medical University. The mice were randomly divided into two groups: Control group (*n* = 3) in which the mice received NSCLC cells without any treatment and ACADL group (*n* = 6) that mice received NSCLC cells overexpressing ACADL. A total of 5 × 10^6^ cells were suspended in 100 µl PBS and inoculated subcutaneously into the right flank of each mice. After 5 days of inoculation, three mice in the ACADL groups were randomly selected for intraperitoneal injection of XMU-MP-1 (1 mg/kg) twice a week for three weeks, while the remaining three were treated with an equal amount of PBS. The mice were monitored and measured every 5 days, and no deaths were observed during this study. Humane endpoints were established to prevent pain or distress in mice, and euthanasia was performed when the tumor volume exceeded 4.2 cm^3^ or when the mice lost more than 20% of their body weight within 1–2 weeks [34]. After 25 days of observation, the subcutaneous tumors were harvested by cervical dislocation under anesthesia with an intraperitoneal injection of 60 mg/kg pentobarbital sodium. Tumor volume was calculated using the formula: volumes = width^2^ × length/2.

### Statistical analysis

Statistical analyses were performed using SPSS software (version 16; SPSS). Data are presented as mean ± S.D. from at least three independent experiments. The comparisons between two groups were conducted with unpaired Student’s *t*‑test. And the comparisons among three or more groups, one‑way ANOVA or two‑way ANOVA analysis followed by Tukey’s multiple comparisons post hoc test was conducted. *P* < 0.05 was considered statistically significant.

## Results

### ACADL downregulation indicates poor prognosis for NSCLC patients

**Fig. 1:**
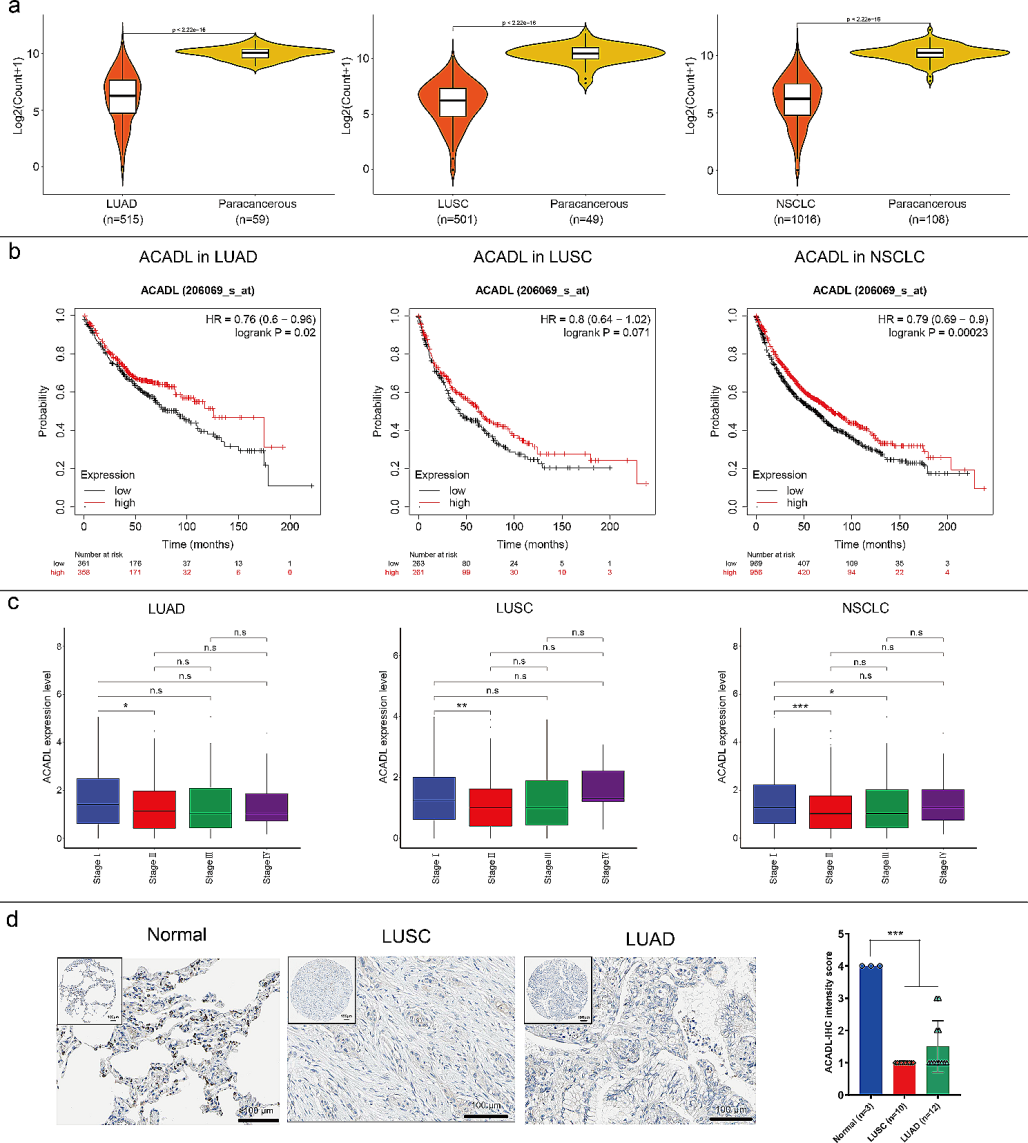
Loss of ACADL indicated a poor prognosis in NSCLC. **a** Compared with paracancerous, ACADL mRNA is down-regulated in NSCLC samples form TCGA database. **b** ACADL expression is positively associated with better prognosis of NSCLC analyzed by Kaplan-Meier plotter. **c** ACADL expressions in different stages of NSCLC. **d.** the represent images for ACADL protein expression in which ACADL is decreased in NSCLC in comparison with paracancerous tissue (Scale bar: 100 μm). *P < 0.05; **P < 0.01; ***P < 0.001. ACADL: Acyl-CoA dehydrogenase long chain. NSCLC: Non-small cell lung cancer. TCGA: The Cancer Genome Atlas

LUSC and LUAD constitute the overwhelming majority of NSCLC cases. As depicted in Fig. 1a, ACADL expression was found to be decreased in both LUAD and LUSC compared to their corresponding paracancerous samples (*P* < 0.001). Moreover, Kaplan-Meier survival analyses revealed a positive correlation between ACADL levels and the prognosis of LUAD (*P* < 0.05, Fig. 1b). While no significant correlation in survival time was observed between LUSC and ACADL (*P* > 0.05), higher expression of ACADL showed a significant improvement in the prognosis of NSCLC (*P* < 0.001, Fig. 1b). In both LUAD and LUSC, ACADL expression in StageII was lower than that in StageI(*P* < 0.05 in LUAD and *P* < 0.01 in LUSC). However, ACADL expression had no significant difference between StageI and StageIII/IV (*P* > 0.05), suggesting ACADL may play a certain protective role in the early progression (from StageII to StageI) of NSCLC (Fig. 1c). Lastly, ACADL protein expression was verified via the LUSC and LUAD clinic tissue in which ACADL expressions in LUAD and LUSC were significantly weaken compared with that in normal lung tissue (Figs. 1d and S1a).

### ACADL inhibited the growth of NSCLC cells in vitro

**Fig. 2:**
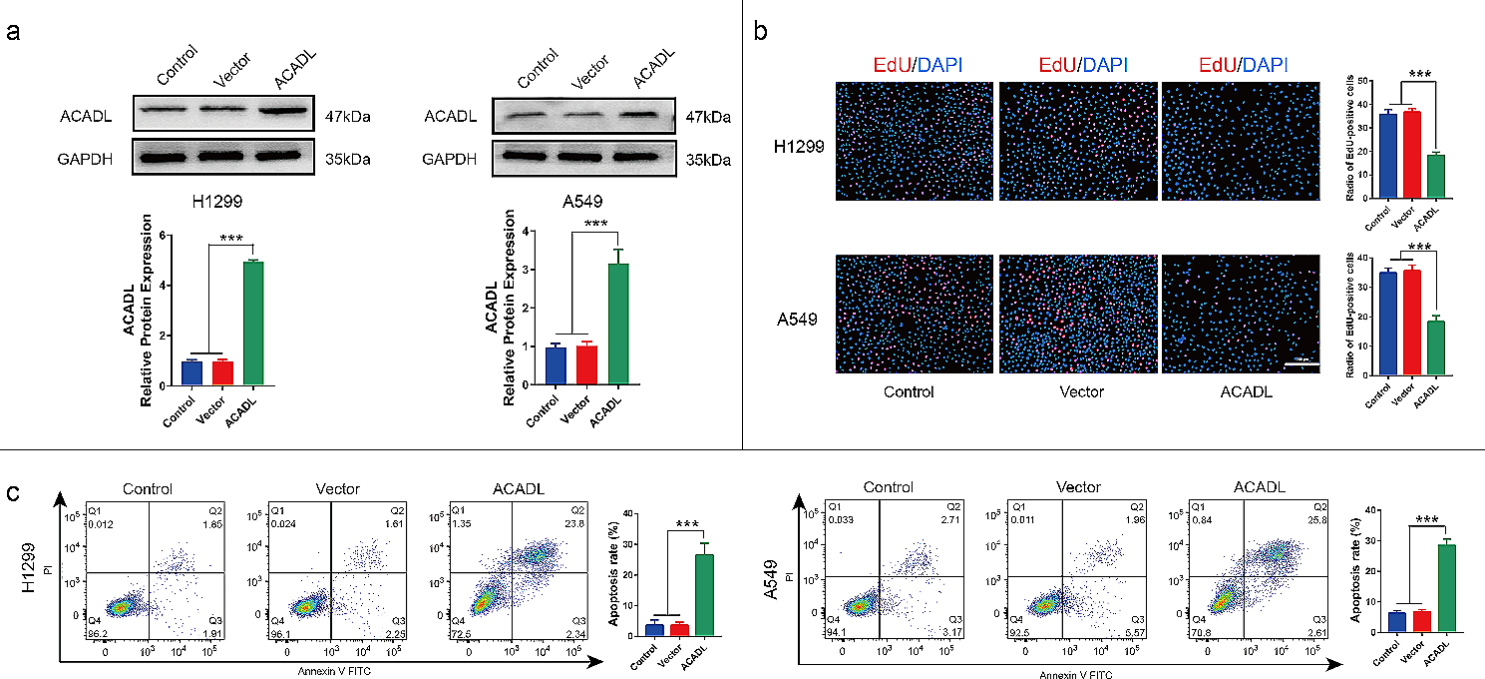
ACADL inhibited proliferation and promoted apoptosis of NSCLC in vitro. **a** ACADL protein expression was increased after ACADL lentiviral vector transfection as the results of western blot. **b.** ACADL overexpression inhibited NSCLC cell proliferation ability verified by EdU staining (Scale bar: 200 μm). **c.** ACADL overexpression promoted NSCLC cell apoptosis analyzed by flow cytometry. ***P < 0.001. ACADL: Acyl-CoA dehydrogenase long chain. NSCLC: Non-small cell lung cancer

To investigate the tumor-suppressive role of ACADL in NSCLC, we first detected the expression of ACADL in four NSCLC cell lines (H292, H1944, H1299 and A549) and ACADL protein expression in H1299 and A549 was lower than that in H292 and H1944 (Fig S1b). We believed that the H1299 and A549 cell lines, due to their lower expression of ACADL, were more suitable for investigating ACADL effect on NSCLC cells. Therefore, H1299 and A549 were selected to construct the stably over-expressing ACADL cell lines. The cell lines were validated by western blot analysis, which showed that the ACADL expression was significantly higher in the ACADL group compared to the Control group (without treatment) or the Vector group (negative control vector treatment) (*P* < 0.001, Fig. 2a).

**Fig. 3:**
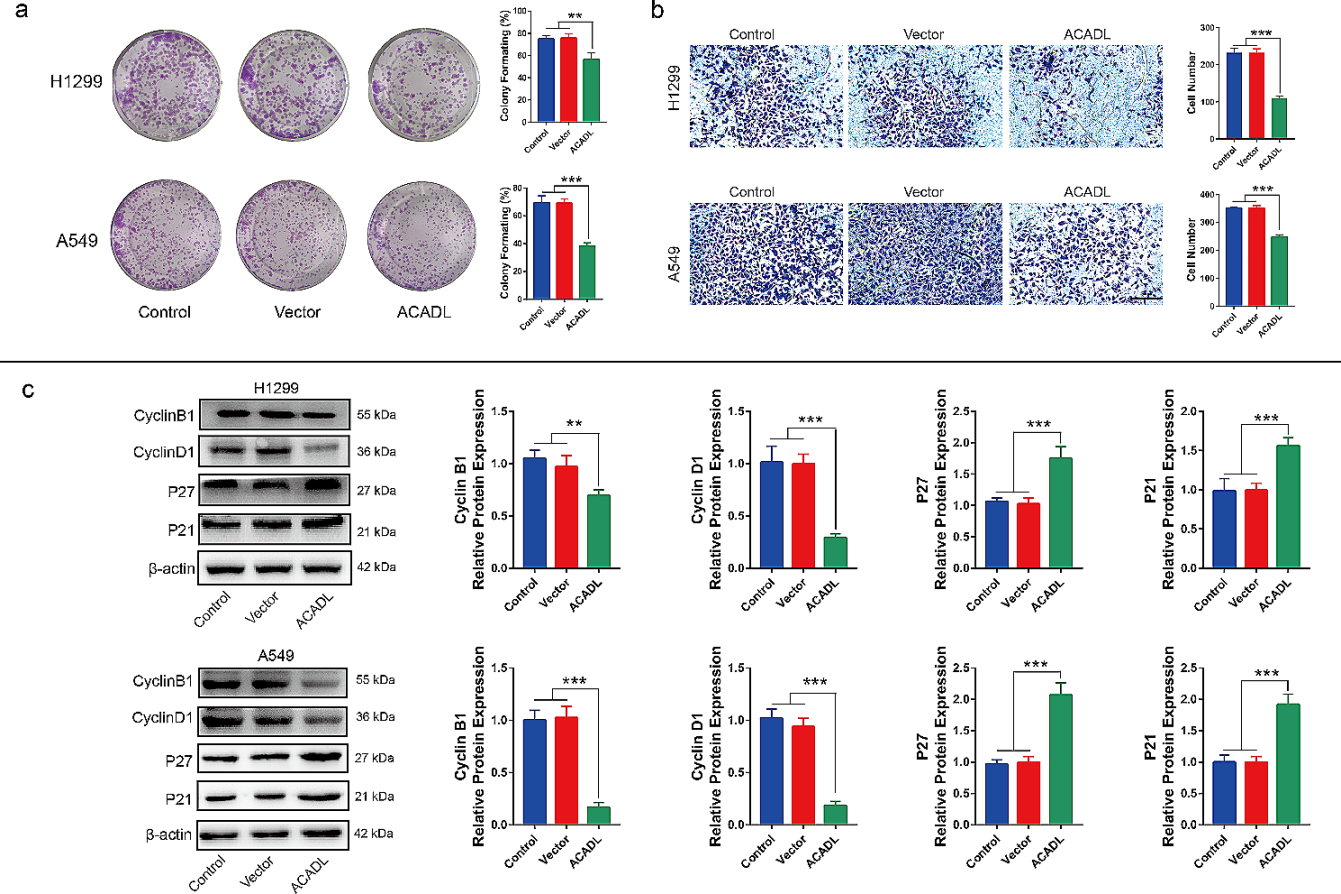
ACADL inhibited the abilities of colony, migration and cellular cycle of NSCLC in vitro. **a** ACADL overexpression reduced the colony ability of NSCLC analyzed via the colony formation assay. **b** ACADL overexpression reduced the invasive cell number according to the transwell assay (Scale bar: 200 μm). **c** Overexpression of ACADL inhibited the protein levels of cyclin B1, cyclin D1, and promote the P27 and P21 via the western blot results. **P < 0.01; ***P < 0.001. ACADL: Acyl-CoA dehydrogenase long chain. NSCLC: Non-small cell lung cancer

As expected, the EdU + cells in ACADL group were reduced by 48.7% in H1299 and 47.4% in A549, compared with that in Control group (*P* < 0.001, Fig. 2b). In addition, in H1299, the apoptosis ability in ACADL group was significantly increased to 26.5% in comparison with its 3.6% in Control group (*P* < 0.001, Fig. 2c). In A549, the apoptosis ability in ACADL group was up to 28.5%, compared with its 6.2% in Control group (*P* < 0.001, Fig. 2c). Subsequently, compared with Control group, the colony number was reduced by 24.9% (*P* < 0.01) and 45.2% (*P* < 0.001) in H1299 and A549 after ACADL overexpression, respectively (Fig. 3a). And the same phenomenon was also observed in the invasiveness in which the invasive cell number was reduced by 52.6% (*P* < 0.001) in H1299 and 31.2% (*P* < 0.001) in A549 after ACADL overexpression (Fig. 3b). Moreover, in H1299, ACADL overexpression could significantly reduce the expressions of Cyclin B1 and Cyclin D1 (positive regulators of the cell cycle) by 33.9% (*P* < 0.01), 71.7% respectively (*P* < 0.001); and increased P27 and P21 (negative regulators of the cell cycle) expressions by 64.5% (*P* < 0.001) and 58.8% (*P* < 0.001, Fig. 3c) [35–37]. In A549, ACADL overexpression significantly reduced the expressions of Cyclin B1 and Cyclin D1 by 83.3% (*P* < 0.01), 81.9% respectively (*P* < 0.001); and increased P27 and P21 expressions by 113.6% (*P* < 0.001) and 91.5% (*P* < 0.001, Fig. 3c). Collectively, these findings demonstrate that ACADL overexpression inhibits the oncogenic properties of NSCLC cells.

### ACADL absence inhibited the growth of NSCLC cells in vitro

Next, to investigate the effect of ACADL deficiency on NSCLC cells, ACADL was knock out in H292 and H1944 due to their high levels of ACADL. Firstly, ACADL expression was significantly decreased in the KO-ACADL group in comparison with Control or KO-SCR groups in both H292 and H1944 (Fig S2a). In addition, ACADL deficiency significantly increased the number of EdU + cells by approximately 1.75 times in H292 and 1.5 times in H1944 (Fig S2b). And the absence of ACADL could significantly reduce the apoptosis rate by 64% in H292 and 61% in H1944 (Fig S2c). As expected, the number of clones in the KO-ACADL group was 68%, 65% higher than that in the Control and KO-SCR groups in H292 and H1944, respectively (Fig S2d). At the same time, ACADL deficiency increased the invasive cell number by 1.4 times in both H292 and H1944 (Fig S2e). ACADL knock out could increase Cyclin B1 and D1 expression by 3.6 times, 2.3 times, reduced P27 and P21 by 42%, 41%, respectively in H292 (Fig S3a). And in H1944, ACADL knock out could increase Cyclin B1 and D1 expression by 4.3 times, 2.1 times, reduced P27 and P21 by 62%, 75%, respectively (Fig S3b). The above results suggested the ACADL absence could inhibit the growth of NSCLC cells in vitro.

### ACADL mediates YAP activation

**Fig. 4:**
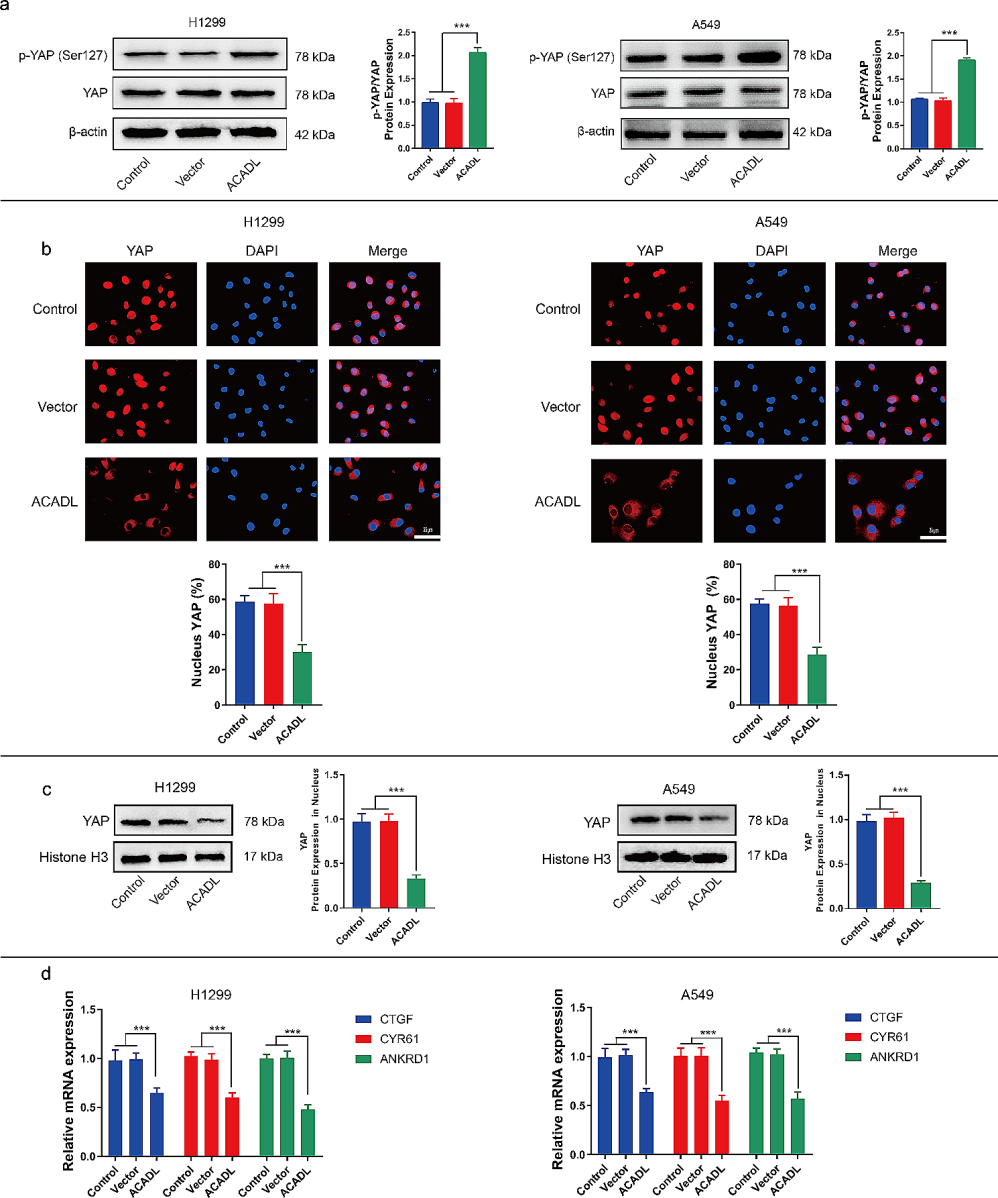
ACADL reduced the YAP activity in NSCLC. **a** The levels of YAP (Ser127) phosphorylation was elevated after ACADL overexpression were detected via western blot results. **b** Representative fluorescence images demonstrated the decrease of YAP nuclear aggregation level after ACADL overexpression (Scale bar: 25 μm). **c** The western blot results revealed the YAP protein level in nucleus was decreased after ACADL overexpression. **d** The qRT-PCR results suggested CTGF, CYR61, and ANKRD1 mRNA in NSCLCs cells were down-regulated after ACADL overexpression. ***P < 0.001. ACADL: Acyl-CoA dehydrogenase long chain. YAP: Yes-associated protein. NSCLC: Non-small cell lung cancer. CTGF: Connective tissue growth factor, CYR61: Cysteine rich angiogenic inducer 61, ANKRD1: Ankyrin repeat domain 1

To investigate the mechanism underlying ACADL-mediated inhibition of cell growth, we examined the activity of the oncogenic transcriptional activator YAP. Phosphorylation of YAP at serine 127 retains it in the cytoplasm, limiting its transcriptional activity and promoting tumor regression [38]. As shown in Fig. 4a, ACADL overexpression increased the YAP phosphorylation level by 109.8% in H1299 (*P* < 0.001) and 79.5% A549 cells (*P* < 0.001), compared to the Control group (Fig. 4a). ACADL knock out could inhibited YAP phosphorylation level by 74%, 63% in H292, H1944, respectively (Fig S4a). At the same, application exogenous ACADL could restore 1.8 times and 1.7 times ACADL expression, compared with KO-ACADL group in both H292 and H1944 cells (Fig S4b).

Immunofluorescence analysis further demonstrated reduced intranuclear YAP levels in ACADL-overexpressing NSCLC cells compared to the Control group (Fig. 4b). The same results were also verified by the expression of YAP in nucleoprotein. The results suggested ACADL overexpression reduced the YAP level by 65.8% in H1299 (*P* < 0.001) and 71.4% in A549 (*P* < 0.001) (Fig. 4c). Furthermore, we assessed the mRNA levels of canonical YAP target genes, such as Ankyrin Repeat Domain 1 (ANKRD1), Cysteine Rich Angiogenic Inducer 61 (CYR61), and Connective Tissue Growth Factor (CTGF). In H1299, the expression levels of ANKRD1, CYR61 and CTGF was reduced by 52.2% (*P* < 0.001), 41.5% (*P* < 0.001), 33.9% (*P* < 0.001) in the ACADL group compared to the Control group (Fig. 4d). In A549, the expression levels of ANKRD1, CYR61 and CTGF was reduced by 45.3% (*P* < 0.001), 45.1% (*P* < 0.001), 35.4% (*P* < 0.001) in the ACADL group compared to the Control group (Fig. 4d).

**Fig. 5:**
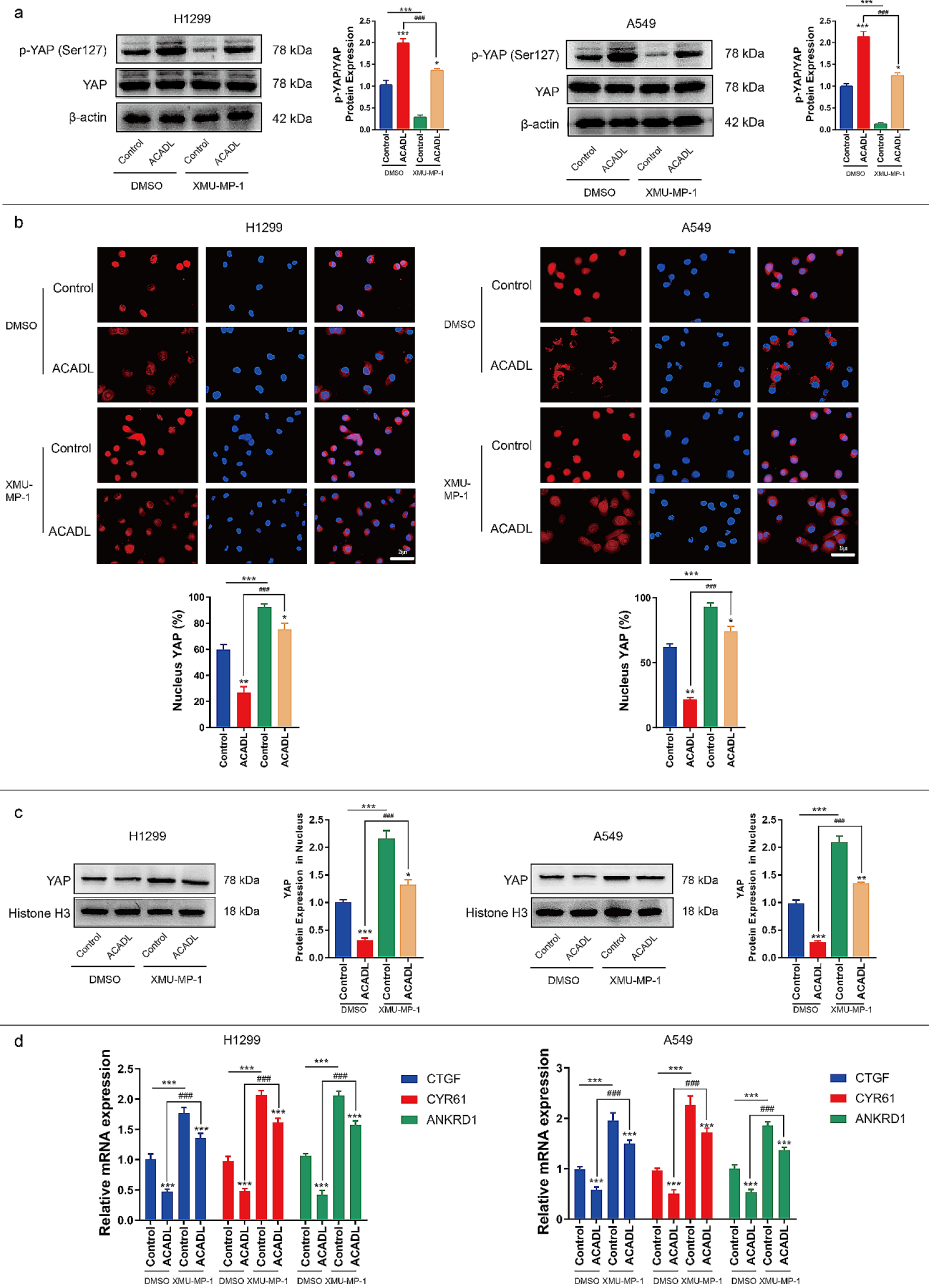
XMU-MP-1 application partly abolished ACADL suppressed function on YAP in NSCLC cells. **a** The western blot results revealed XMU-MP-1 application in ACADL overexpression cell could partly rescue the level of YAP phosphorylation. **b** Representative fluorescence images demonstrated XMU-MP-1 application in ACADL overexpression cell could increase the YAP nuclear aggregation level (Scale bar: 25 μm). **c** The western blot results revealed XMU-MP-1 application in ACADL overexpression cell could rescue the level of YAP in nucleus. **d** The qRT-PCR results suggested XMU-MP-1 application in ACADL overexpression cell could rescue the mRNA expressions of CTGF, CYR61, and ANKRD1. * vs. Control with DMSO group; * *P* < 0.05; ** *P* < 0.01; *** *P* < 0.001. ^#^ vs. ACADL with DMSO group; ^###^*P* < 0.001. ACADL: Acyl-CoA dehydrogenase long chain. YAP: Yes-associated protein. NSCLC: Non-small cell lung cancer. CTGF: Connective tissue growth factor, CYR61: Cysteine rich angiogenic inducer 61, ANKRD1: Ankyrin repeat domain 1

Moreover, treatment with XMU-MP-1, a YAP activator, partly reversed ACADL-induced YAP phosphorylation by 31.8% in H1299 (*P* < 0.001) and by 42.1% in A549 (*P* < 0.001) (Fig. 5a), led to nuclear accumulation of YAP (Fig. 5b). As expected, XMU-MP-1 treatment also significantly promoted YAP nuclear transfer by 4.3 times in H1299 (*P* < 0.001) and 4.9 times in A549 (*P* < 0.001) compared with that in ACADL group (Fig. 5c). At the same time, in H1299, XMU-MP-1 application also promoted ANKRD1, CYR61, CTGF expression by 3.7 times (*P* < 0.001), 3.3 times (*P* < 0.001), 2.9 times (*P* < 0.001) in comparison with ACADL groups (Fig. 5d). Similarly, in A549, XMU-MP-1 application significantly increased ANKRD1, CYR61, CTGF expression by 2.6 times (*P* < 0.001), 3.4 times (*P* < 0.001), 2.6 times (*P* < 0.001) in comparison with ACADL groups (Fig. 5d). These findings indicate that ACADL overexpression promotes YAP phosphorylation, restricts its nuclear location, and attenuates the transcriptional activity of YAP in NSCLC cells.

### YAP activation reduces ACADL-mediated cell growth inhibition

**Fig. 6:**
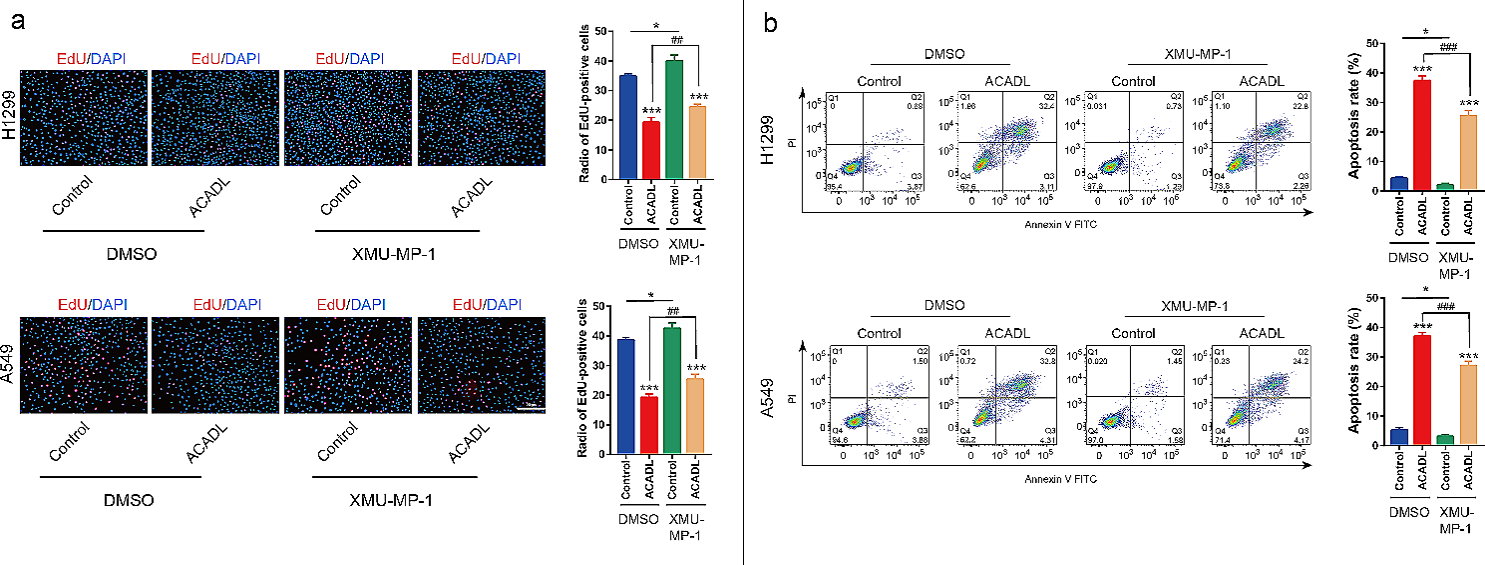
YAP reactivation decreased ACADL-mediated NSCLC cells growth. **a** XMU-MP-1 application in ACADL overexpression cell could partly restore the NSCLC proliferative ability according to the EdU staining results (Scale bar: 200 μm). **b** XMU-MP-1 application in ACADL overexpression cell could partly abolish the pro-apoptotic effect of ACADL on NSCLC cell according to the flow cytometry results. * vs. Control with DMSO group; * *P* < 0.05; *** *P* < 0.001. ^#^ vs. ACADL with DMSO group; ^##^*P* < 0.01; ^###^*P* < 0.001. ACADL: Acyl-CoA dehydrogenase long chain. YAP: Yes-associated protein. NSCLC: Non-small cell lung cancer

**Fig. 7:**
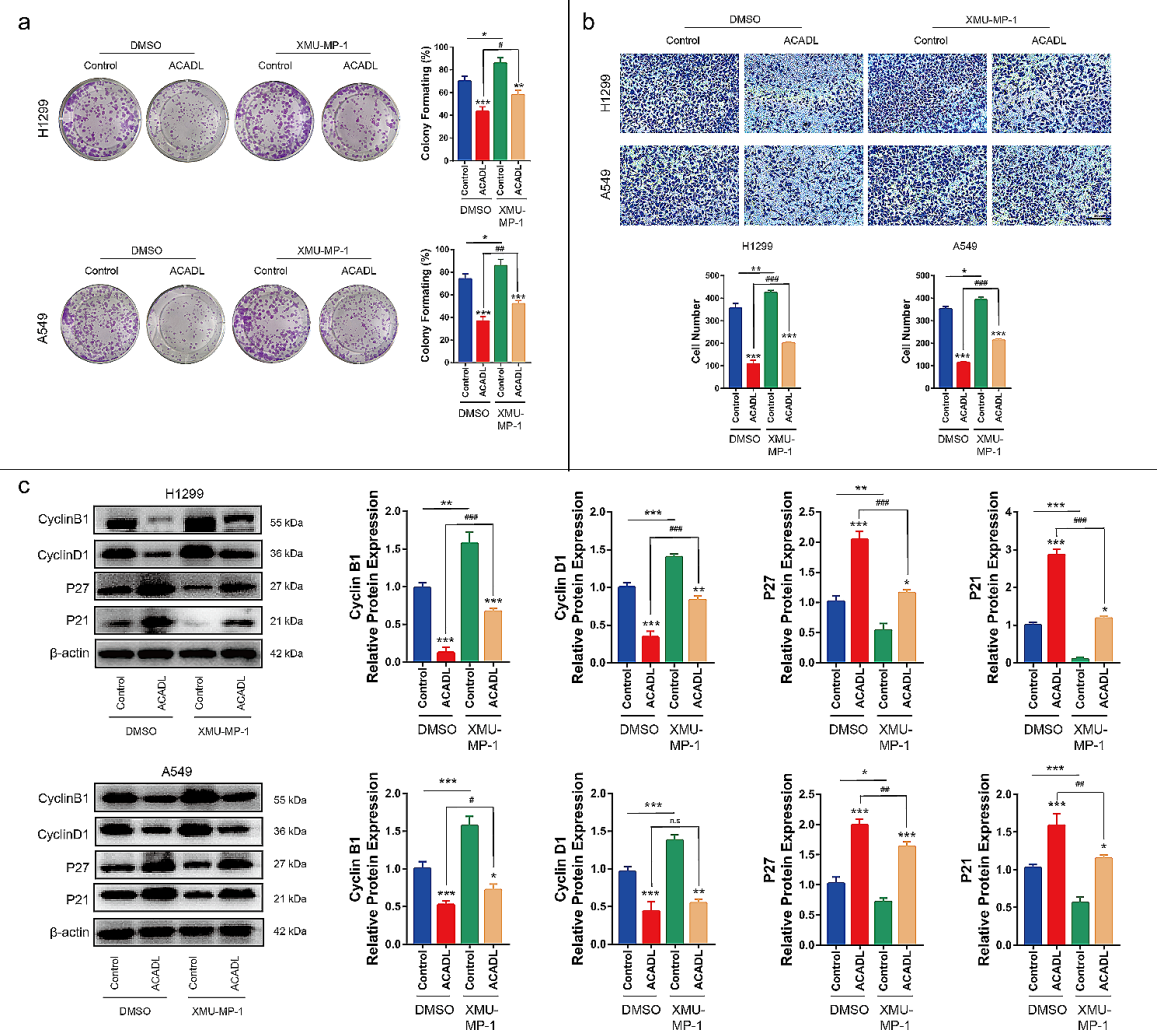
YAP reactivation weaken ACADL-mediated colony, migration inhibition and cycle arrest of NSCLC in vitro. **a** XMU-MP-1 application in ACADL overexpression cell could partly rescue the NSCLC colony ability according to the colony formation assay. **b** XMU-MP-1 application in ACADL overexpression cell could partly rescue the NSCLC invasive ability according to the transwell assay (Scale bar: 200 μm). **c** XMU-MP-1 application in ACADL overexpression cell could partly rescue the expression of cyclin B1, cyclin D1, abolished the positive effect on P27 and P21 of ACADL as the results of western blot. * vs. Control with DMSO group; * *P* < 0.05; ** *P* < 0.01; *** *P* < 0.001. ^#^ vs. ACADL with DMSO group; ^#^*P* < 0.05; ^##^*P* < 0.01; ^###^*P* < 0.001. ACADL: Acyl-CoA dehydrogenase long chain. YAP: Yes-associated protein. NSCLC: Non-small cell lung cancer

To further confirm the association between YAP and ACADL-mediated NSCLC cell growth, we investigated the effect of a YAP activator on the oncogenic properties of ACADL-overexpressing NSCLC cells. Initially, treatment with XMU-MP-1 increased the percentage of EdU^+^ cells by 26.7% in H1299 (*P* < 0.01), 32.2% in A549 (*P* < 0.01) compared with that ACADL group (Fig. 6a). And the apoptosis level was reduced by 31.9% in H1299 (*P* < 0.001) and 27.2% in A549 (*P* < 0.001) after XMU-MP-1 treatment in comparison with that in ACADL group (Fig. 6b). Additionally, XMU-MP-1 partially reversed the decreased colony-forming ability observed in the ACADL overexpression group. Specifically, the colony number was increased by 33.8% in H1299 (*P* < 0.05) and 41.8% in A549 (*P* < 0.01) after XMU-MP-1treatment (Fig. 7a). Moreover, the transwell migration assay demonstrated that XMU-MP-1 treatment increased the invasive ability of ACADL overexpressing group by 1.9 times in both A549 (*P* < 0.001, Fig. 7b). Similarly, XMU-MP-1 administration increased the expression of CyclinB1 and Cyclin D1 by 4.2 times, 2.4 times respectively; inhibited P27 and P21 expression by 0.6 times, 0.4 times in ACADL overexpressing H1299 (*P* < 0.001, Fig. 7b). In ACADL overexpressing A549, XMU-MP-1 application increased the expression of cyclinB1 and Cyclin D1 by 1.4 times (*P* < 0.05), 1.3 times respectively (*P* > 0.05); inhibited P27 and P21 expression by 0.8 times (*P* < 0.01), 0.7 times (*P* < 0.01) (Fig. 7b). These results suggest that ACADL-induced YAP phosphorylation is essential for the inhibitory effect of ACADL on NSCLC cell proliferation.

### ACADL inhibits NSCLC cell growth by regulating YAP activity in vivo

**Fig. 8:**
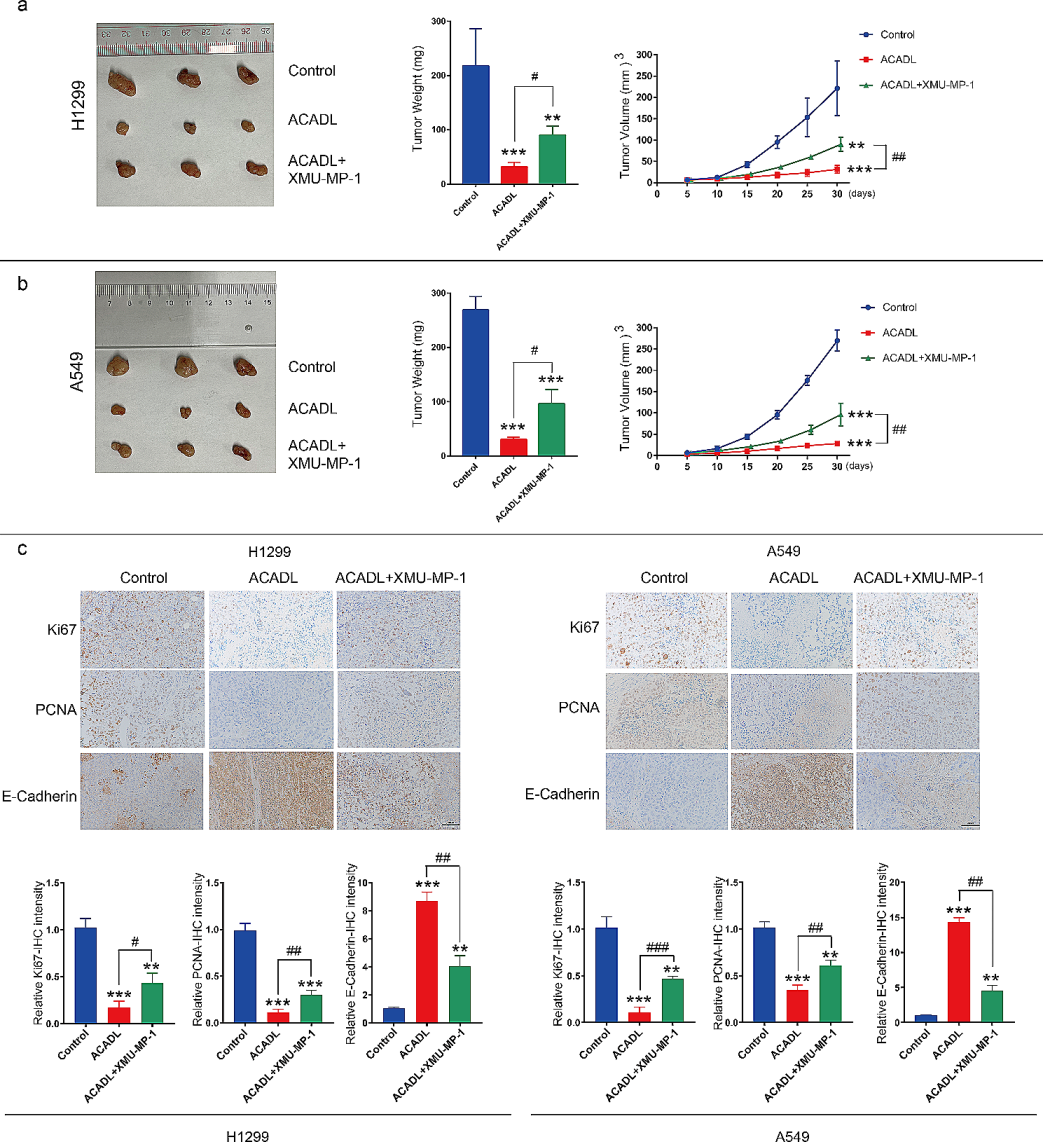
ACADL inhibited growth of NSCLC cells in vivo by inhibiting the activation of YAP. **a**, **b** ACADL could promote the growth of NSCLC cells in vivo, while XMU-MP-1 application could partially inhibit the growth promoting effect of ACADL on NSCLC cells. **c** The expression of Ki67 and PCNA was significantly decreased in ACADL overexpression tumors. XMU-MP-1 application could partly rescue the phenomena. E-Cadherin expression was higher in ACADL group and was weaken after XMU-MP-1 application. * vs. Control group; **P < 0.01; ***P < 0.001. ^#^ vs. ACADL group; ^#^*P* < 0.05; ^##^*P* < 0.01. ACADL: Acyl-CoA dehydrogenase long chain. YAP: Yes-associated protein. NSCLC: Non-small cell lung cancer

Based on the aforementioned findings demonstrating the significant inhibition of NSCLC cell growth by ACADL in vitro, we proceeded to investigate the effect of ACADL on neoplasm growth in vivo using a mouse xenograft tumor model. In both H1299 and A549, the volume of tumor formation in ACADL groups was about 0.1 times (*P* < 0.001) compared to that in Control group. And XMU-MP-1 effectively counteracted the inhibitory effect of ACADL by 0.3 times on the tumor volume (*P* < 0.01) (Fig. 8a and b). As a typical tumor suppressor factor, P53 was significantly upregulated in the ACADL group according to IHC results, but could be partially reversed by the application of XMU-MP-1 (Fig. 8c). Moreover, IHC analysis revealed a notable decrease in the expression of proliferative proteins Ki67 and PCNA in ACADL overexpressing tumor tissues, which was partially reversed in the XMU-MP-1 treatment group (Fig. 8c and d). Additionally, loss of E-cadherin expression is associated with epithelial-mesenchymal transition (EMT) and tumor metastasis. We observed robust E-cadherin expression in ACADL overexpressing tumor tissues, which was partially attenuated by XMU-MP-1 treatment (Fig. 8c). These results further support the inhibitory role of ACADL in the proliferation and growth of NSCLC cells in vivo through its regulation of YAP activity.

## Discussion

Lung cancer, being a prevalent and highly aggressive malignancy, ranks among the leading causes of death worldwide [39, 40]. Although the application of various chemoradiotherapy [41], gene targeting drugs [42], and advanced immune targeted drugs [43] has significantly improved the prognosis of NSCLC, there are still some patients who cannot benefit from advanced above strategies because of individual tumor heterogeneity. Only by continuously deepening our understanding of NSCLC can we help develop more effective treatment strategies. In the present study, we found significant downregulation of ACADL expression by comparing the expression profiles of NSCLC and adjacent tumors, and demonstrated that overexpression of ACADL can inhibit tumor progression in vitro and transplanted tumor models. More importantly, we found that ACADL exerts an anti-tumor effect by promoting the phosphorylation of YAP and limiting its nuclear translocation.

Initially identified for its role in lipid and energy metabolism, ACADL has been implicated in various metabolic disorders, including central obesity, cardiovascular disease, diabetes, and nonketotic hypoglycemia. Subsequent studies revealed a relationship between ACADL and tumor progression in various human cancers, such as HCC [20], melanoma [44], breast cancer [45], ESCC [18], and prostate carcinoma [19]. However, the role of ACADL can vary across different tumor types. For instance, ACADL functions as an oncogene and is up-regulated in ESCC, while it is down-regulated in hepatocellular carcinoma and exhibits a suppressive effect on tumor cells. As the leading cause of cancer-related deaths worldwide, although many pathological molecular mechanisms related to lung cancer, especially NSCLC, have been discovered and successful immunotherapies against PD-1 have been developed, there are still many NSCLC patients who cannot benefit from them. Therefore, exploring the function and role of ACADL in NSCLC can help further improve the molecular theory of NSCLC and provide reliable theoretical references for future targeted gene therapy. In the present study, analysis of the TCGA database demonstrated decreased expression of ACADL in both LUAD and LUSC. Consistently, higher ACADL expression was significantly associated with better prognosis in NSCLC. Moreover, ACADL overexpression in NSCLC cell lines effectively inhibited cell proliferation, weakened invasion capability, promoted apoptosis, and induced cycle arrest of NSCLC cells. Furthermore, the inhibitory effect of ACADL on NSCLC cells was confirmed in a xenografted tumor model, where ACADL overexpressing cells formed tumors at a significantly slower rate compared to the Control group in vivo. Collectively, these findings support the notion that ACADL functions as a tumor suppressor in NSCLC.

YAP, along with TAZ, plays a crucial role in regulating organ growth and cell plasticity during animal development and regeneration [46]. Excessive activation of YAP has been reported as a significant marker in lung, colorectal, and gastric cancers [47–49]. YAP promotes tumor progression by initiating the transcription of canonical YAP target genes (ANKRD1, CYR61, CTGF) through nuclear translocation [20]. Phosphorylation of YAP at serine127 sequesters it in the cytosol and limits its transcriptional activity and positive carcinogenic effect [50]. ANKRD1 localizes to the nucleus and is a component a complex called Titin that carries cancer-associated “driver” mutations [51, 52]. Moreover, ANKRD1 has been confirmed to be associated with chemotherapy resistance in various tumors, including ovarian cancer and lung cancer [53, 54]. Similarity, abnormal activation of CYR61 has also been detected in various tumors, such as colorectal cancer [55], breast cancer [56], and cervical cancer [57]. In terms of mechanism, CYR61 may accelerate tumor progression by intervening in the tumor microenvironment [58], and this effect has also been proven to rely on YAP activation [59]. CTGF, companied with YAP and CYR61 can be highly expressed in tamoxifen resistant breast cancer, leading to poor prognosis in patients [56], suggesting that CTGF is an important component of YAP in promoting tumor development. Additional, CTGF has also been detected in more tumor types, and some reports suggest that CTGF may be a new therapeutic target for tumors [60].

In our study, we observed that ACADL overexpression in NSCLC cells resulted in YAP phosphorylation and reduced nuclear translocation. Furthermore, ACADL overexpression significantly inhibited the transcription of canonical genes like ANKRD1, CYR61 and CTGF. In addition, YAP is involved in the progression of NSCLC by transferring into the nucleus and participating in the transcription of various oncogenic genes. ACADL was able to inhibit the movement of YAP into the nucleus, thereby exerting an inhibitory effect on tumor growth. Additionally, the YAP agonist XMU-MP-1 promoted YAP nuclear translocation [61]. Application of XMU-MP-1 reversed the cytosol accumulation and phosphorylation of YAP induced by ACADL overexpression and partially mitigated the repression of ACADL on cell cycle progress, cell proliferation, and cellular invasion in NSCLC. Consistently, similar experimental results were observed in the xenograft tumor model where XMU-MP-1 partially counteracted the inhibitory effect of ACADL on tumor growth rate.

In summary, our findings demonstrate the tumor-suppressive role of ACADL in NSCLC by enhancing the nuclear translocation of YAP. However, our study has some limitations and shortcomings. For example, we only established the involvement of ACADL in NSCLC progression, and further animal experiments are needed to verify whether ACADL could serve as a therapeutic target for NSCLC. Moreover, although ACADL exhibited differential expression in NSCLC and adjacent normal tissues, it remains to be confirmed whether ACADL can serve as an early diagnostic marker for NSNCL to enable early screening. Considering these limitations, future research and applications of ACADL should focus on exploring its potential as an early screening marker for NSCLC and as an effective target for gene and immunotherapy.

## Conclusion

Reduced expression of ACADL in NSCLC correlated with a poor prognosis. Increased ACADL levels were found to suppress cell proliferation and invasion by reducing YAP activity. These findings suggest that ACADL may serve as a novel target in the progression of NSCLC and holds promise as a potential therapeutic target for NSCLC treatment.

### Electronic supplementary material

Below is the link to the electronic supplementary material.

Supplementary Material 1

## Data Availability

The datasets collected and analyzed during the current study are available from the corresponding author upon reasonable request.
