# Supplementary material for: ACADL-YAP axis activity in non-small cell lung cancer carcinogenicity
Source: Cancer Cell Int. 2024 Feb 24;24:86. doi: 10.1186/s12935-024-03276-7 (PMC10894480; doi:10.1186/s12935-024-03276-7)
Supplement: Supplementary file 1 — Supplementary Material 1 [file 12935_2024_3276_MOESM1_ESM.docx]

**
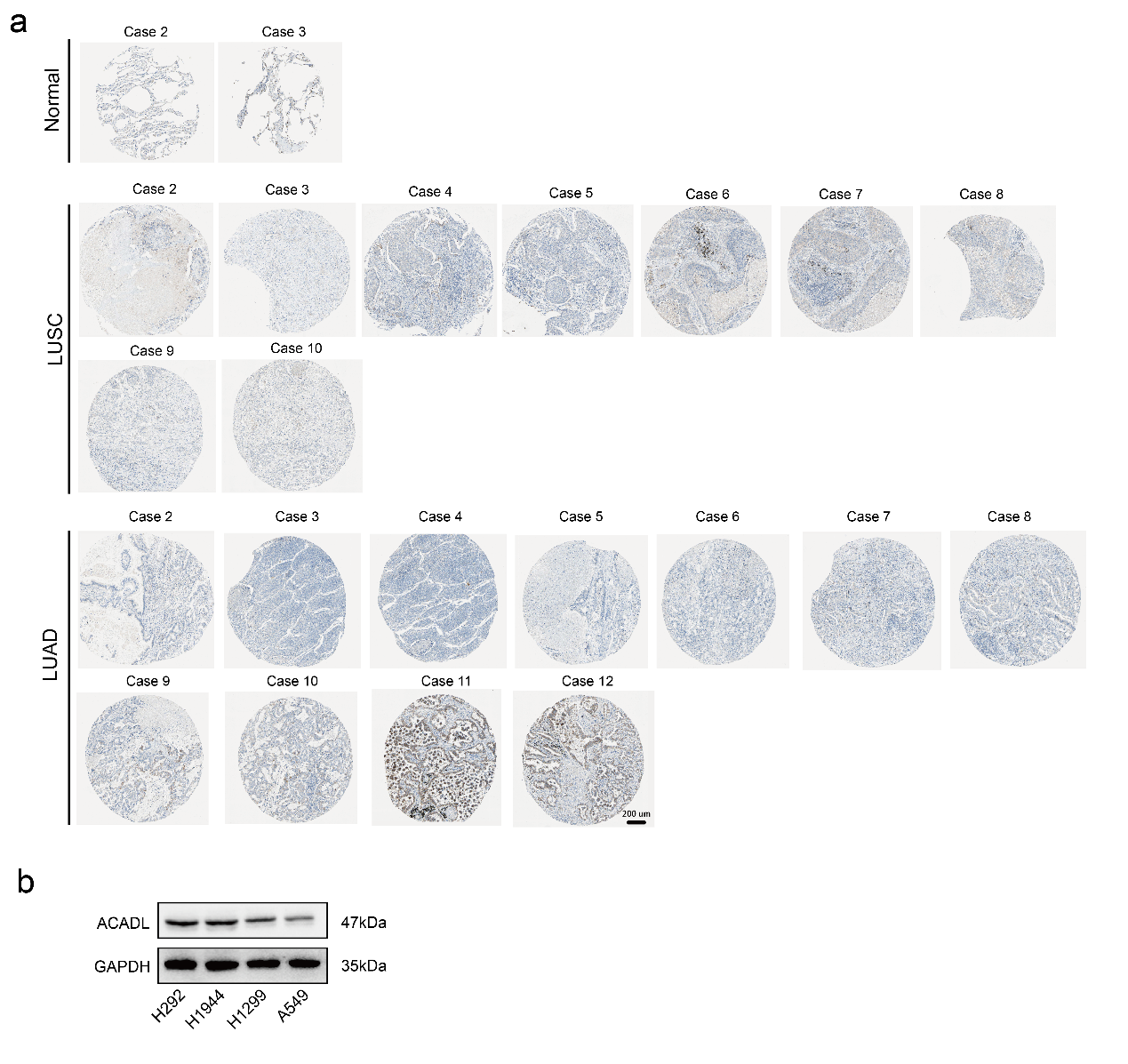
Figure S1. ACADL expression in normal lung tissue, NSCLC samples and NSCLC cell lines. a.** All the normal and NSCLC samples collected from The Human Pathology Atlas database (scale bar = 200 µm). **b.** The protein expression ACADL of some NSCLC cell line.

**Figure S2. The absence of ACADL accelerated the malignant phenotype of NSCLC cells. a.**
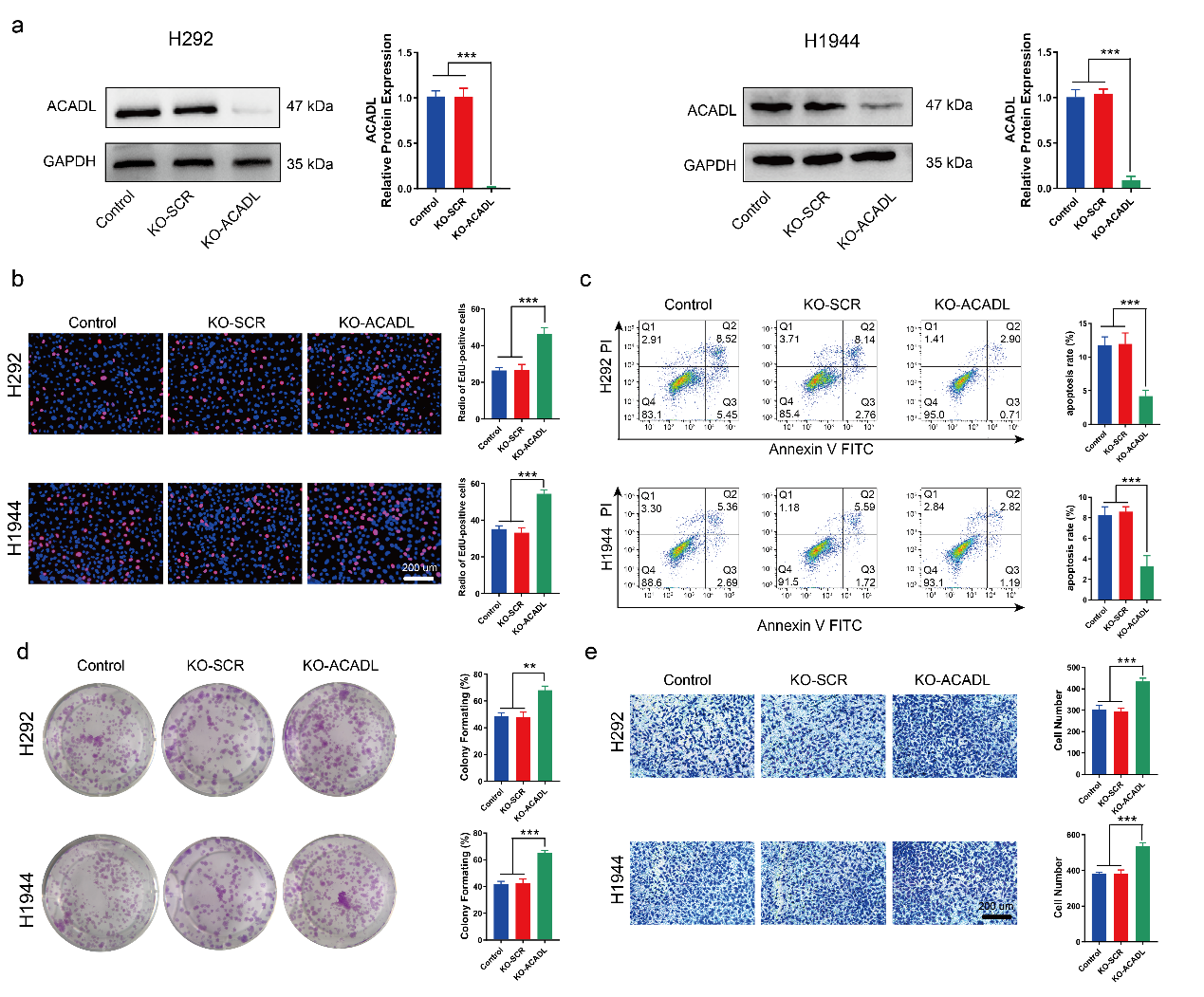
 ACALD protein expression was significantly decreased in KO-ACADL group, compared with Control and KO-SCR groups according to the western blot assay. ACALD knock out promoted the proliferation ability of NSCLC cells verified by the EdU assay **(b, scale bar = 200 µm)**, inhibited the apoptosis as the results of flow cytometry **(c)**, enhanced the colony **(d)** and invasion **(e, scale bar = 200 µm)** abilities according to colony formation and transwell assays. NSCLC: Non-small cell lung cancer. ACADL: Acyl-CoA dehydrogenase long chain. KO-SCR: Knock out the scrambled sequence. KO-ACADL: Knock out the ACADL. ***P*<0.01, ****P*<0.001.


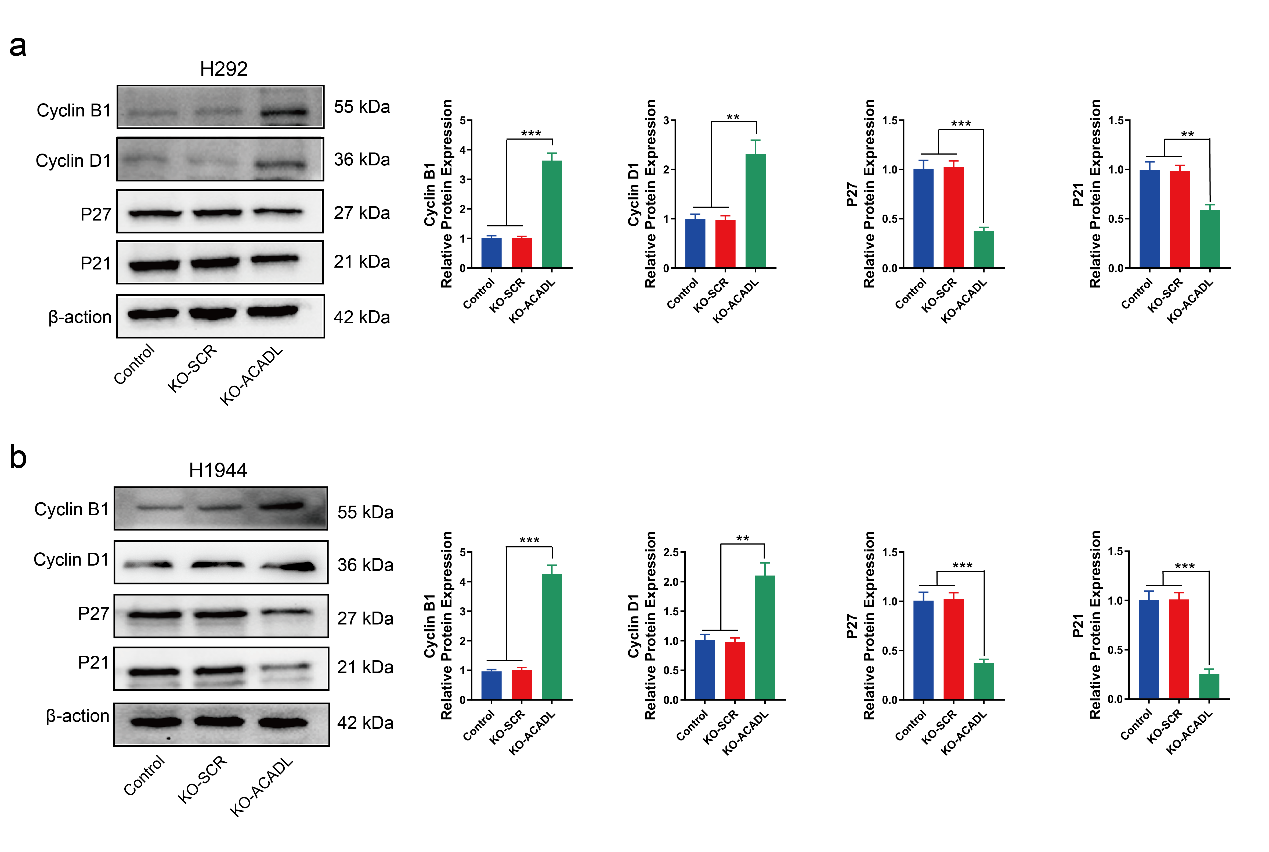
**Fig S3. The absence of ACADL accelerated the cell cycles of NSCLC cells. a.** In H292 cells and H1944 cells **(b)**, ACADL knock out significantly up-regulated Cyclin B1, Cyclin D1 expression; down-regulated P27 and P21 expressions, compared with Control and KO-SCR groups according to the western blot results. NSCLC: Non-small cell lung cancer. ACADL: Acyl-CoA dehydrogenase long chain. KO-SCR: Knock out the scrambled sequence. KO-ACADL: Knock out the ACADL. ***P*<0.01, ****P*<0.001.


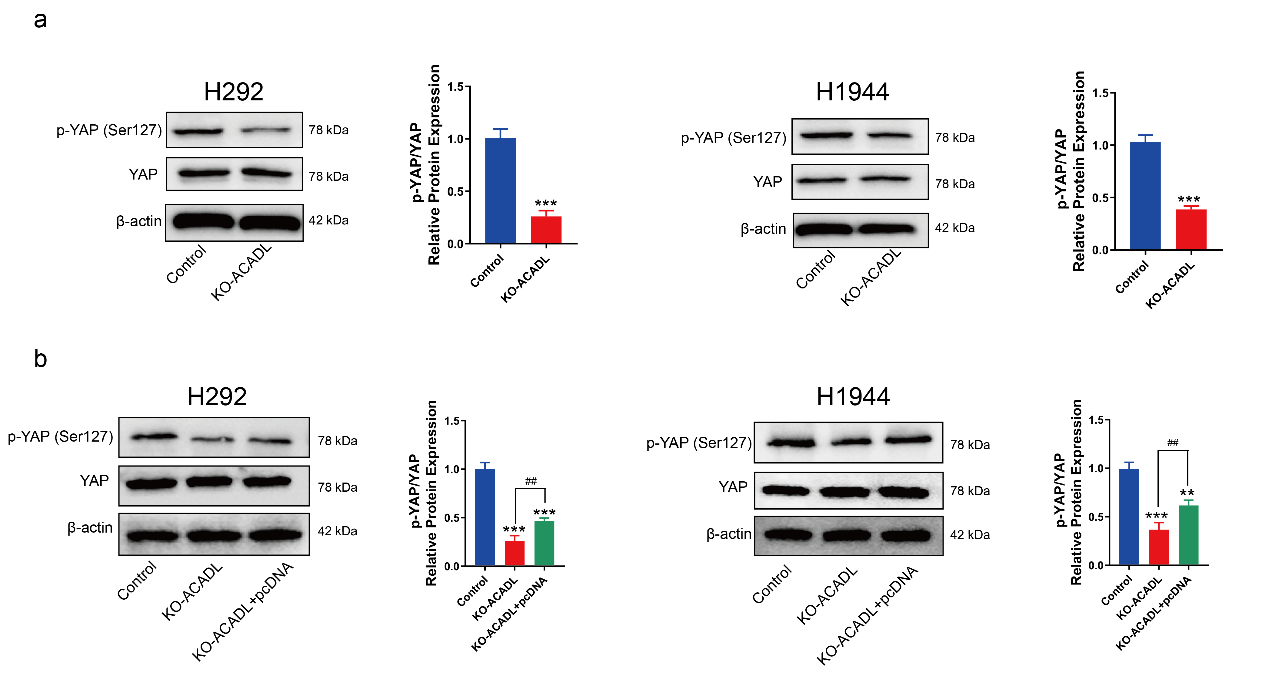
**Fig S4. The absence of ACADL decreased the YAP phosphorylation level of NSCLC cells. a.** ACADL knock out significantly decreased the level of YAP phosphorylation as the results of western blot. **b.** Exogenous ACADL could partially restore the phosphorylation level of ACADL according to the results of western blot. NSCLC: Non-small cell lung cancer. ACADL: Acyl-CoA dehydrogenase long chain. YAP: Yes-associated protein. KO-ACADL: Knock out the ACADL. KO-ACADL+pcDNA: KO-ACADL cells with ACADL overexpression plasmid. ****P*<0.001 vs Control. ^##^*P*<0.01 vs KO-ACADL group.
